# Structural and molecular characterization of lopinavir and ivermectin as breast cancer resistance protein (BCRP/ABCG2) inhibitors

**DOI:** 10.17179/excli2023-6427

**Published:** 2023-11-14

**Authors:** Julia de Paula Dutra, Gustavo Scheiffer, Thales Kronenberger, Lucas Julian Cruz Gomes, Isadora Zanzarini, Kelly Karoline dos Santos, Arun K. Tonduru, Antti Poso, Fabiane Gomes de Moraes Rego, Geraldo Picheth, Glaucio Valdameri, Vivian Rotuno Moure

**Affiliations:** 1 Graduate Program in Pharmaceutical Sciences, Laboratory of Cancer Drug Resistance, Federal University of Parana, Curitiba, PR, Brazil; 2 School of Pharmacy, Faculty of Health Sciences, University of Eastern Finland, P.O. Box 1627, FI-70211 Kuopio, Finland; 3 (a) Department of Internal Medicine VIII, University Hospital Tuebingen, Otfried-Müller-Strasse 14, Tuebingen DE 72076, Germany, (b) Department of Pharmaceutical and Medicinal Chemistry, Institute of Pharmaceutical Sciences, Eberhard-Karls-Universität, Tuebingen, Auf der Morgenstelle 8, 72076 Tuebingen, Germany, (c) Cluster of Excellence iFIT (EXC 2180) “Image-Guided and Functionally Instructed Tumor Therapies”, University of Tuebingen, 72076 Tuebingen, Germany, (d) Tuebingen Center for Academic Drug Discovery & Development (TüCAD2), 72076 Tuebingen, Germany; 4 Graduate Program in Pharmaceutical Sciences, Federal University of Parana, Curitiba, PR, Brazil

**Keywords:** ABCG2, ABCG2 inhibitors, drug repositioning, lopinavir, ivermectin, molecular modelling

## Abstract

A current clinical challenge in cancer is multidrug resistance (MDR) mediated by ABC transporters. Breast cancer resistance protein (BCRP) or ABCG2 transporter is one of the most important ABC transporters implicated in MDR and the use of inhibitors is a promising approach to overcome the resistance in cancer. This study aimed to characterize the molecular mechanism of ABCG2 inhibitors identified by a repurposing drug strategy using antiviral, anti-inflammatory and antiparasitic agents. Lopinavir and ivermectin can be considered as pan-inhibitors of ABC transporters, since both compounds inhibited ABCG2, P-glycoprotein and MRP1. They inhibited ABCG2 activity showing IC_50_ values of 25.5 and 23.4 µM, respectively. These drugs were highly cytotoxic and not transported by ABCG2. Additionally, these drugs increased the 5D3 antibody binding and did not affect the mRNA and protein expression levels. Cell-based analysis of the type of inhibition suggested a non-competitive inhibition, which was further corroborated by *in silico *approaches of molecular docking and molecular dynamics simulations. These results showed an overlap of the lopinavir and ivermectin binding sites on ABCG2, mainly interacting with E446 residue. However, the substrate mitoxantrone occupies a different site, binding to the F436 region, closer to the L554/L555 plug. In conclusion, these results revealed the mechanistic basis of lopinavir and ivermectin interaction with ABCG2.

See also the Graphical abstract(Fig. 1).

## Introduction

Most cancer treatment protocols include chemotherapy, however, the development of resistance is responsible for the lower objective response rate observed with the classical protocols (Gottesman et al., 2016[18]). The development of simultaneous cross-resistance to a wide of structurally unrelated drugs is called multidrug resistance (MDR) (Szakács et al., 2006[54]). The MDR phenomenon in cancer can be categorized as intrinsic (pre-existing) or acquired, triggered by drug exposure (Gottesman, 2002[16]). Different strategies to overcome MDR in cancer have been proposed, albeit it remains an important oncological challenge, since several cellular mechanisms are involved in MDR, including the inactivation of the drug, enhanced DNA repair, mutations or altered expression levels of the biological target, drug compartmentalization, altered mitochondria, failure of programmed cell death and overexpression of ABC transporters (Gottesman et al., 2002[17]; Hall et al., 2009[22]; Szakács et al., 2014[53]).

The human genome encodes 48 ABC proteins, most of them transporters that promote the efflux of substrates mediated by ATP binding and hydrolysis (Dean et al., 2001[11]). ABC transporters show important physiological functions, pumping xenobiotics out of cells to protect them from damage, illustrating their localization in sanctuaries sites in our body, such as the blood-brain barrier (BBB), blood-testis barrier and blood-placental barrier (Robey et al., 2018[47]). In cancer cells, three ABC transporters are considered most relevant, with undoubted association with chemotherapeutic treatment failure: P-glycoprotein (P-gp or MDR1, encoded by *ABCB1*), multidrug resistance-associated protein 1 (MRP1, encoded by *ABCC1*) and breast cancer resistance protein (BCRP, encoded by *ABCG2*) (Gottesman et al., 2002[17]; Szakács et al., 2006[54]). 

ABCG2 was discovered in 1998 by three independent research groups, receiving different names based on the biological model, including BCRP, because of its identification in a breast cancer cell line (Doyle et al., 1998[13]), MXR from resistance caused by mitoxantrone (Miyake et al., 1999[35]) and ABCP, due their presence in placenta (Allikmets et al., 1998[5]). Today, ABCG2 has used to unequivocally announce this transporter. Considering that efflux inhibition consists of the most promising strategy to overcome MDR mediated by ABC transporters, more than a hundred ABCG2 inhibitors have already been identified (Zattoni et al., 2022[71]). The first described ABCG2 inhibitor was the fungal toxin fumitremorgin C (FTC) (Rabindran et al., 1998[45]). FTC was used as a scaffold for analogues synthesis like Ko143, which is considered a ABCG2 reference inhibitor (Allen et al., 2002[4]). The current list of ABCG2 inhibitors includes several classes of compounds, including chalcones (Valdameri et al., 2012[62]), indeno[1,2-*b*]indoles (Kita et al., 2021[31]), stilbenes (Valdameri et al., 2012[64]), tetrahydroquinoline/4,5-dihydroisoxazole hybrids (Vesga et al., 2021[65]) and chromones (Valdameri et al., 2012[63]), that can be classified as specific toward ABCG2, dual or pan-inhibitors of ABC transporters (Zattoni et al., 2022[71]).

Drug repurposing is an interesting and attractive strategy for the rapid identification of ABCG2 inhibitors from existing medicines, that possess distinct molecular targets and have already been identified safe therapeutic agents in humans (Shim and Liu, 2014[50]; Zattoni et al., 2022[71]). Several classes of drugs already were screened as ABCG2 inhibitors, including antibiotics, antifungals, anti-HIV, calcium channel blockers, glucocorticoids and tyrosine kinase inhibitors (Boumendjel et al., 2011[8]; Juvale and Wiese, 2015[28]; Zattoni et al., 2022[71]). Among the few potent ABCG2 inhibitors that show IC_50_ values in the nanomolar range, some were identified by drug repurposing, such as tivozanib, fostamatinib, ponatinib and febuxostat (Zattoni et al., 2022[71]). In this work, we initially tested the ability of antiviral, anti-inflammatory and antiparasitic drugs that were used to treat COVID-19 to inhibit ABCG2 transporter. Lopinavir and ivermectin have been described as ABC transporters inhibitors (Bierman et al., 2010[7]; Telbisz et al., 2021[58]), but the mechanism of interaction was not characterized, which is important to delineate future pre-clinical experiments.

## Material and Methods

### Materials

Mitoxantrone, rhodamine 123, GF120918 (Elacridar), Ko143, hydrocortisone, prednisolone, dexamethasone, ivermectin, lopinavir, hydroxychloroquine, chloroquine and oseltamivir and MTT were purchased from Sigma-Aldrich. Hoechst 33342 and TRIzol were purchased from Invitrogen. All other reagents were commercial products of the highest available purity.

### Cell cultures

Human HEK293 parental cells (wild-type) and HEK293 cells stably transfected with ABCG2 (HEK293-*ABCG2*), mouse NIH3T3 parental cells (wild-type) and NIH3T3 cells stably transfected with P-gp (NIH3T3-*ABCB1*), hamster BHK21 parental cells (wild-type) and BHK21 cells stably transfected with MRP1 (HEK293-*ABCC1*) were provided by Dr Attilio Di Pietro (IBCP, Lyon, France). All cells were maintained in Dulbecco's Modified Eagle's Medium (DMEM high glucose) supplemented with 10 % fetal bovine serum (FBS), 1 % penicillin/streptomycin, and with 0.75 mg/mL G418 (HEK293-*ABCG2*), 60 ng/mL colchicine (NIH3T3-*ABCB1*) or 0.1 mg/mL methotrexate (BHK21-*ABCC1*) at 37 °C in 5 % CO_2_ atmosphere.

### Inhibition assay

The ability of drugs to inhibit the transport function of ABC proteins was evaluated using fluorescent substrates by flow cytometry. Cells were aliquoted at a density of 1.0 x 10^5^ cells/tube. Cells were exposed to fluorescent substrates (Hoechst 33342 at 3 µM, rhodamine 123 at 10 µM, calcein-AM at 0.15 µM and mitoxantrone from 2.5 to 25 µM) with or without drugs at different concentrations, and incubated at 37 °C in 5 % CO_2_ for 45 min. The cells were centrifuged (2,000 x g for 5 min) and resuspended with 300 µL of cold phosphate buffer saline (PBS) and kept on ice until flow cytometry analysis. Intracellular substrate fluorescence data were acquired using a FACS Celesta (equipped with three lasers: 355, 405 and 488 nm) or a FACS Calibur (equipped with two lasers: 488 and 635 nm) flow cytometer. At least 10,000 events were collected, and the median fluorescence intensities were used for the calculations. The inhibition percentage was calculated using parental cells or a reference inhibitor to achieve 100 % of inhibition. In all experiments, at least three independent replicates were used and IC_50_ values were calculated by using GraphPad prism software version 6.01.

### Cell viability assay

Cells were seeded (2.0 x 10^4^ cells/well) into a 96 wells plate and incubated for 24 h to the attachment. The cells viability was evaluated by MTT (3-[4,5-dimethylthiazol-2-yl]-2,5 diphenyl tetrazolium bromide) assay. Cells were treated with increasing concentrations of drugs for 72 h. After this period, the medium was discarded and the cells monolayer was washed with PBS (100 μL), followed by incubation at 37 °C with MTT solution (100 μL of solution 0.5 mg/mL in PBS) for 4 h. Then, the solution was discarded, and the formazan crystals dissolved with 100 μL of ethanol/DMSO (1:1). The absorbance was measured using a microplate reader at 595 nm (Bio-Rad iMark).

### Conformational antibody binding (5D3) assay

The effect of drugs on the binding of a conformational antibody was determined by flow cytometry. HEK293-*ABCG2* cells were cultivated at 37 °C in 5 % CO_2_ until approximately 90 % of confluence, then detached and separated in tubes with 5 x 10^5^ cells/tube. Cells were centrifuged at 2,000 x g for 5 min and the supernatant was discarded. The cell pellet was suspended in PBS (100 µL) containing 40 µg/mL of BSA. Samples were incubated with inhibitors for 10 minutes at 37 °C. After this period, the primary antibody anti-human ABCG2 clone 5D3 (BD Pharmingen - dilution 1:100) was added to each sample and incubated for 30 min at 37 °C. Cells were centrifuged and the supernatant was discarded. Cells were suspended in PBS (100 µL) and the secondary antibody was added (anti-mouse PE, Abcam - dilution 1:200). The samples were incubated at 37 °C for 30 min, centrifuged, and the cell pellet was suspended in 300 µL of PBS. Data were recorded by flow cytometry. At least 10,000 events were collected.

### RT-qPCR

Total RNA was obtained of HEK293-*ABCG2 *cells from tissue flasks-25 cm^2 ^(at approximately 90 % of confluence) after treatment for 72 h with lopinavir (6.25 µM) and ivermectin (1.56 µM). The total RNA isolation was performed using TRIzol (Invitrogen) protocol according to the manufacturer's instructions. RNA concentration was quantified by absorbance using the NanoDrop™ spectophotometer and the integrity was evaluated by 1 % agarose gel electrophoresis. RNA was stored at -80 °C. Two micrograms of total RNA were reverse transcribed using High Capacity cDNA Reverse Transcription Kit (Applied Biosystems) according to the manufacturer's instructions, and the resulting cDNA was stored at -20 °C. Using cDNAs as the template, quantitative real-time PCR was performed using the SYBR Green PCR Master Mix (Applied Biosystems) in a 7500™ Real-Time PCR Detection System (Applied Biosystems). A dissociation cycle was performed after each run to check for non-specific amplification or contamination. The mRNA expression levels were normalized using the geNorm 3.4 software, and the corresponding housekeeping gene expression levels. Sets of specific primers were designed using Primer designing tool - NCBI and validated through BLAST and BLAT, and their respective sequences are shown in Table 1(Tab. 1) (References in Table 1: Ali et al., 2015[3]; Jacob et al., 2013[24]; Lemma et al., 2016[33]; Potashnikova et al., 2015[44]; Saidova et al., 2018[48]; Sharan et al., 2015[49]; Zhang et al., 2017[73]). Relative expression levels were estimated using the method described by Pfaffl (2001[43]).

### Western blot

Protein was obtained from HEK293-*ABCG2 *cells from tissue flasks-25 cm^2 ^(at approximately 90 % of confluence) after treatment for 72 h with lopinavir (6.25 µM) and ivermectin (1.56 µM) using 200 µL of RIPA+ buffer and 2 µL of 0.5 M EDTA pH 8. Protein quantification was performed by Bradford and 40 µg was separated by gel electrophoresis (8 % polyacrylamide). Proteins were transferred to a PVDF membrane using a semi-dry transference system (GE Healthcare). The membrane was cut in two parts guided by the PageRuler™ Prestained protein ladder. The upper part of the membrane was incubated overnight (4 °C) with ABCG2 antibody (BXP-21, diluted 1:500) in TBST buffer (1.5 M NaCl, 0.5 mM Tris and 0.1 % Tween 20) containing 1 % powdered milk. The lower part of the membrane was incubated overnight (4 °C) with GAPDH antibody (mAB, diluted 1:5000) in TBST buffer containing 1 % powdered milk. The membranes were washed in TBST, and incubated with anti-mouse IgG HRP antibody, diluted 1:2,000 in TBST containing 1 % powdered milk at room temperature for 1 hour. After this incubation, the membranes are washed with TBST. ECL Plus kit substrate (GE Healthcare) was added to the membranes and exposed to a Amersham Hyperfilm (GE Healthcare) for 10 seconds.

### Protein selection and preparation for in silico studies

ABCG2 structure with mitoxantrone was retrieved from Protein Data Bank (PDB), PDB ID 6VXI, resolution 3.7 Å (Orlando and Liao, 2020[41]). The structure was prepared and minimized by adding hydrogens, adjusting protonation states (pH 7.4) of amino acids, and fixing missing side-chain atoms using Maestro PrepWizard (version 2021.4). The missing loops between K46 and E60 (N-terminal domain or NTD), S302-P327 (NTD) and G354-Y369 (connecting the transmembrane bundle and the NTD) were generated using Prime (Jacobson et al., 2004[25]) and the final structure encompassed from A35 to S655.

### Molecular docking

Subsequently, the structures had only polar hydrogens maintained and were converted to pdbqt format using the Autodock Tools 1.5.6 (Morris et al., 2009[38]). The ligand structures for ivermectin, lopinavir and mitoxantrone were downloaded from ZINC Database (Sterling and Irwin, 2015[51]), taken into the Avogadro software and subjected to a geometry pre-optimization using the Auto Optimization tool (MMFF94s force field) (Halgren and Nachbar, 1996[21]; Halgren, 1999[20]), followed by visual inspection to ensure that there were no errors. Using Avogadro, the molecule file was prepared for a second geometry optimization in MOPAC2016 (Stewart, 2016[52]), with the semi-empirical quantum PM7 method. At the end of this step, the files were converted to the pdbqt format, also in Autodock Tools, ensuring that all torsions were set to active. For docking, the Autodock Vina 1.2.3 (Eberhardt et al., 2021[14]) was used, in which a grid box was delimited, based on the central region in which the co-crystallized ligand was originally detected. The mitoxantrone + lopinavir (MTX + LPV) and mitoxantrone + ivermectin (MTX + IVT) docking was performed sequentially, using the 6VXI + MTX docked structure for the inhibitors. The atomic coordinates of the gridbox centroid for all docking experiments were defined as: X = -0.181; Y = -0.222; Z = 0.571, with a 40 Å distance on all three axes. The exhaustiveness parameter was set to 75 and the maximum number of results (poses) to twenty, with a maximum allowed variation of 2 kcal/mol from the first to the last conformation. After, the process was performed with all molecules (substrate and inhibitors), resulting poses served as a starting point for molecular dynamics simulations (MD).

### Molecular dynamics simulations

The minimized structures were submitted to MD simulation for further refinement, using a previously published protocol (Zattoni et al., 2022[72]). Selected docking poses were further validated by MD simulation, where ligand stability within the proposed pocket and its interactions were evaluated. The MD simulations were carried out using the Desmond engine (Bowers et al., 2006[9]) with the OPLS4 force-field (Lu et al., 2021[34]). The simulated system encompassed the protein-ligand complex, a predefined water model (TIP3P;´Jorgensen et al., 1983[27]) as a solvent, POPC membranes (automatically positioned according to the alpha-helices), and counterions (Na^+^ or Cl^-^ adjusted to neutralize the overall system charge). The system was treated in an orthorhombic box with periodic boundary conditions specifying the shape and the size of the box as 10x10x13 Å distance from the box edges to any atom of the protein. RESPA integrator time steps of 2 fs for bonded and near, and 6 fs for non-bonded terms far were applied. Short-range coulombic interactions were performed using a time step of 1 fs and a cut-off value of 9.0 Å, whereas long-range coulombic interactions were handled using the Smooth Particle Mesh Ewald (PME) method (Darden et al., 1993[10]). Standard Desmond relaxation protocol was employed. Simulations were run in the NPT ensemble, with a temperature of 310 K (Nosé-Hoover thermostat) and pressure of 1.01325 bar (Martyna-Tobias-Klein barostat). MD trajectories were visualized, and figures were produced using PyMOL v.2.5 (Schrödinger LCC, New York, NY, USA). At least three independent simulations were performed for each ligand, being 200 ns for inhibitors and 500 ns for mitoxantrone.

### Trajectory analyses and MM/GBSA

Protein-ligand interactions were determined using the simulation event analysis pipeline implemented in Maestro (Maestro v2021.4). Distance calculations were performed employing the Maestro event analysis tool (Schrödinger, LLC, New York, NY). The molecular mechanic energies with generalized Born and surface area continuum solvation (MM/GBSA) were calculated with Prime (Jacobson et al., 2004[25]) thermal MM/GBSA script provided by Schrödinger. Each 5^th^ frame of MD was used for MM/GMBSA calculations. Trajectories were clustered according to the ligand's RMSD values in order to select relevant conformations for discussion and figures, using the trj_cluster.py script provided by Schrödinger. Figures were generated using PyMOL v2.5 (Schrödinger, LLC, New York, NY).

## Results and Discussion

### Identification of lopinavir and ivermectin as ABCG2 inhibitors

In order to identify ABCG2 inhibitors, a repurposing drug strategy utilizing a cell-based model was used. All the eight drugs were selected based on the fact that they were used as therapeutic alternatives for COVID-19, regardless their efficacy. Hydrocortisone, prednisolone, dexamethasone, ivermectin, lopinavir, hydroxychloroquine, chloroquine and oseltamivir were tested to inhibit the activity of ABCG2 in stably transfected HEK293-*ABCG2* cells overexpressing the ABCG2 transporter. This initial screening was performed by flow cytometry using Hoechst 33342 as a fluorescent substrate of ABCG2, and all drugs were essayed at 10 and 100 µM. Only lopinavir and ivermectin inhibited ABCG2 activity (Figure 2A(Fig. 2)). Both drugs produced a mild inhibition effect at 10 µM, about 25 %. However, a complete inhibition (100 %) was observed at 100 µM (Figure 2A and B(Fig. 2)). The ABCG2 inhibition caused by lopinavir (Weiss et al., 2007[69]; Bierman et al., 2010[7]) and ivermectin (Jani et al., 2011[26]) was already reported, and most recently, this inhibition effect was confirmed, showing IC_50_ values (compound concentrations giving a half-maximal inhibition) of ABCG2 inhibition of 13.1 and 3.1 µM, for lopinavir and ivermectin, respectively (Telbisz et al., 2021[58]). Here, we observed IC_50_ values of inhibition of 23.4 and 25.5 µM for lopinavir and ivermectin, respectively (Figure 2C and D(Fig. 2)). The differences among the IC_50_ values could be associated with the substrate used in each study. Telbisz *et al* have used PhenGreen (PG)-AM as substrate of ABCG2 (Telbisz et al., 2021[58]), while we used Hoechst 33342. 

**Figure 1:**
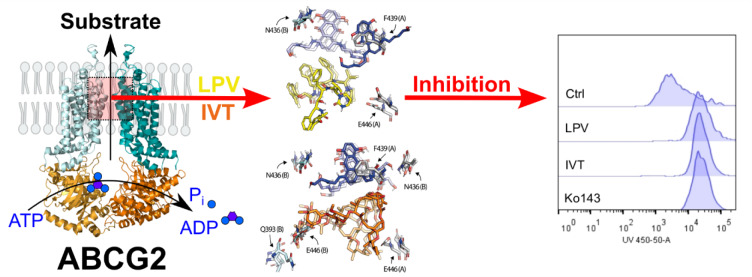
Graphical abstract

**Figure 2:**
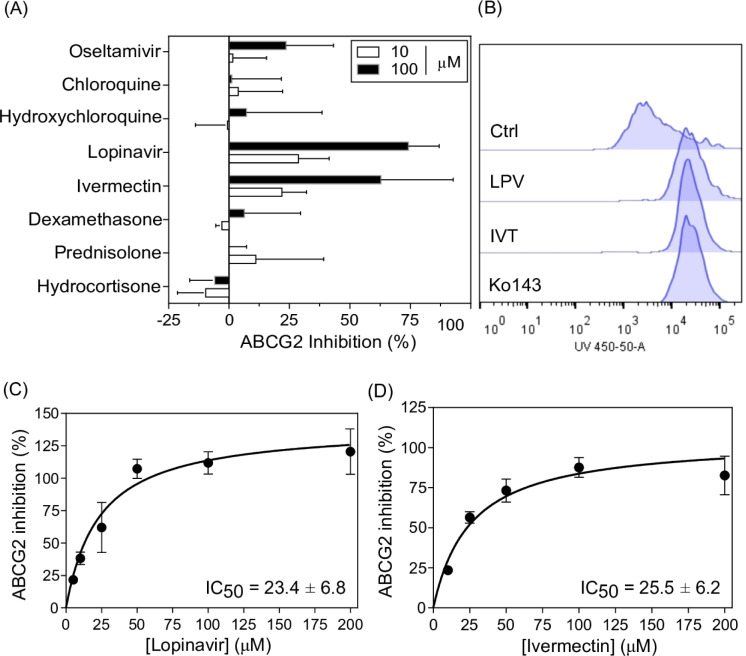
ABCG2 inhibition by flow cytometry. (A) Screening of drugs: hydrocortisone, prednisolone, dexamethasone, ivermectin, lopinavir, hydroxychloroquine, chloroquine and oseltamivir as ABCG2 inhibitors. Drugs were tested at 10 and 100 µM on HEK293-*ABCG2* cells by flow cytometry using Hoechst 33342 as substrate at 3 µM. Ko143 at 1 µM was used as reference inhibitor (100 % of inhibition). Data represent the mean ± SD of at least three independent experiments. (B) Representative histograms of different conditions, including control (Ctrl) with Hoechst 33342 (3 µM) alone and together with lopinavir (100 µM), ivermectin (100 µM) and Ko143 (1 µM). (C) Lopinavir and (D) ivermectin IC_50_ curves of ABCG2 inhibition. Data represent the mean ± SD of at least three independent experiments.

We also investigated the inhibition effect of these eight drugs on P-gp and MRP1 using stably transfected NIH3T3-*ABCB1* cells overexpressing P-gp and BHK21-*ABCC1* cells overexpressing MRP1, respectively. In this case, rhodamine 123 was used as substrate of P-gp and calcein-AM was used as substrate of MRP1. Lopinavir and ivermectin acted as P-gp (see supplementary information Figure 1) and MRP1 (see supplementary information Figure 2) inhibitors. The inhibition at 10 µM was higher for P-gp than for MRP1. Interestingly, the inhibition caused for both drugs at 10 µM on MRP1 was similar to the observed toward ABCG2 (~ 25 %). Both drugs completely inhibited P-gp and MRP1 at 100 µM. Using calcein-AM (Telbisz et al., 2021[58]) or doxorubicin (Bierman et al., 2010[7]) as substrate, ivermectin and lopinavir were also able to inhibit P-gp and MRP1. It was recently described that hydroxychloroquine at concentrations higher than 10 µM inhibits the P-gp activity, without effect on ABCG2 activity (Weiss et al., 2020[68]). Here, we observed that both chloroquine and hydroxychloroquine inhibited the P-gp activity at 100 µM (see supplementary information Figure 1) without effect on ABCG2 (Figure 2A(Fig. 2)). In general terms, despite the search for specific inhibitors, the identification of dual- and pan-inhibitors is attractive (Zattoni et al., 2022[71]). In fact, the clinic trials failure of specific P-gp inhibitors was partially attributed to the overlap of transported substrates among the ABC transporters (Tamaki et al., 2011[56]; Robey et al., 2018[47]). Taken together, we confirmed that lopinavir and ivermectin act as pan-inhibitors of ABC transporters at 100 µM (Figure 2A(Fig. 2), see supplementary information Figures 1 and 2).

### High cytotoxicity and absence of ABCG2-mediated transport of lopinavir and ivermectin

**Figure 3:**
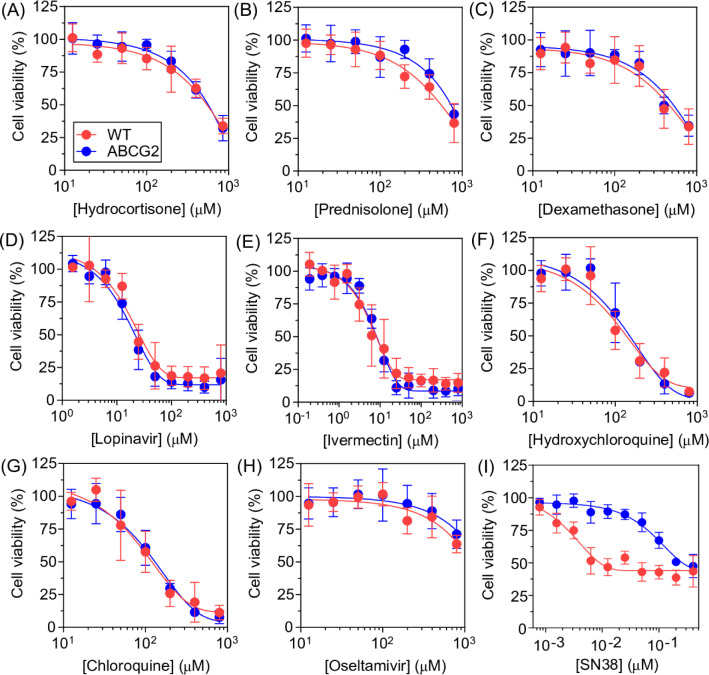
Cytotoxicity and absence of transport mediated by ABCG2 transporter. MTT cell viability assay was performed on HEK293 cells (WT) in red and HEK293-*ABCG2* cells (ABCG2) in blue after 72 hours of treatment with (A) hydrocortisone, (B) prednisolone, (C) dexamethasone, (D) lopinavir, (E) vermectin, (F) hydroxychloroquine, (G) chloroquine, (H) oseltamivir, and (I) SN38. Drugs were tested at different concentrations, as indicated in the graphs and the data represent the mean ± SD of at least three independent experiments. Cells treated with the vehicle (DMSO or H_2_O) were considered 100 % of viable cells.

To further investigate the interaction of the eight drugs with ABCG2, a cell viability assay was performed using HEK293 cells and transfected cells overexpressing ABCG2 (HEK292-*ABCG2*). Many classes of ABCG2 inhibitors also are recognized as ABCG2 substrates, and this effect can be initially investigated by a cell viability assay (Zattoni et al., 2022[71]). However, this approach is only useful for cytotoxic drugs. A lower cytotoxic effect triggered by drugs on cells overexpressing the ABC transporter than on the parental cell line suggests a transport. Since SN38 (the active metabolite of irinotecan) is a substrate of ABCG2, this drug was used as control. As shown in Figure 3(Fig. 3), SN38 was transported by ABCG2, showing a lower cytotoxic effect on cells overexpressing ABCG2. All drugs decreased the cell viability after 72 hours of exposure. Some of these drugs, such as oseltamivir showed a very low cytotoxic effect, decreasing the cell viability only in concentrations higher than 200 µM (Figure 3H(Fig. 3)). In sharp contrast, lopinavir and ivermectin were highly cytotoxic, decreasing the cell viability at 12 and 3 µM, respectively (Figure 3D and E(Fig. 3)). Interestingly, the two drugs that inhibited ABCG2 transport activity also were the most cytotoxic agents. 

The cytotoxic effect is an important parameter to be considered during the identification of ABCG2 inhibitors since many potent inhibitors did not follow pre-clinical studies due to their high intrinsic cytotoxicity (Boumendjel et al., 2011[8]; Zattoni et al., 2022[71]). To correlate the inhibition potency and cytotoxicity, the inhibitors were compared based on their therapeutic rate (IG_50_/IC_50_), where the IG_50_ value is the concentration that decreases 50 % cell viability. In this way, compounds that present a high therapeutic rate (TR), such as chromone 6g (MBL-II-141), that showed a TR of 2000 (Valdameri et al., 2012[63]), are considered promising to be explored in pre-clinical models and clinical trials studies (Zattoni et al., 2022[71]). Here, the calculated TR for lopinavir and ivermectin were 0.64 and 0.26, respectively (see supplementary information Table 1). These very low TR values indicate that both compounds were highly cytotoxic and probably the ABCG2 inhibition effect might not be validated in animal models. However, considering a drug repurposing strategy, another parameter that should be considered is the drug plasma concentration. The highest plasma concentrations described for lopinavir and ivermectin were 15 µM (https://www.accessdata.fda.gov/drugsatfda_docs/label/2007/021226s018lbl.pdf) and 53 nM (https://www.accessdata.fda.gov/drugsatfda_docs/label/2009/050742s026lbl.pdf), respectively. Thus, the clinical use of ivermectin as an ABCG2 inhibitor is impracticable, however, the IC_50_ value of ABCG2 inhibition of lopinavir is in the same order magnitude of the plasma concentrations (µM range). Considering that the most promising ABCG2 inhibitor identified by the drug repurposing strategy is the febuxostat (Miyata et al., 2016[36]; Toyoda et al., 2019[60]), showing a IC_50_ of 27 nM and plasma concentration of 90 nM, its use in clinical dose possibly inhibits ABCG2 (Toyoda et al., 2019[60]). In accordance, we found that ABCG2 is possibly inhibited by lopinavir in clinical dose.

Regarding the cytotoxic profile of the eight drugs comparing both cell lines, HEK293 and HEK293-*ABCG2*, no difference was evidenced (Figure 3(Fig. 3)). These results suggest that none of these drugs are recognized as substrates of ABCG2 transporter. Our data further confirmed that hydroxychloroquine and lopinavir are not substrate for ABCG2 (Agarwal et al., 2007[2]; Bierman et al., 2010[7]; Weiss et al., 2020[68]). The absence of transport mediated by the target transporter is a desirable feature of inhibitors, which shows that lopinavir and ivermectin chemical structures promising scaffolds for the rational design of more potent ABCG2 inhibitors.

**Figure 4:**
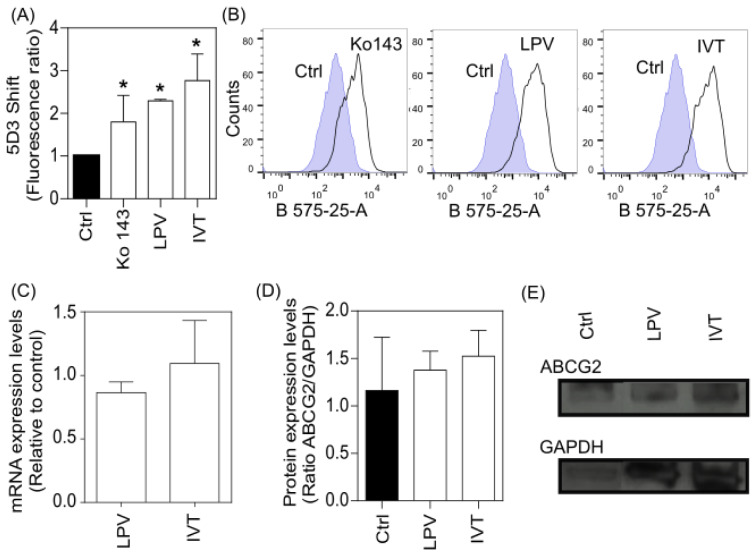
ABCG2 inhibition by flow cytometry. (A) Conformational 5D3 antibody binding. The data was normalized by the untreated control. *Significant difference (*p* > 0.05) according to Kruskal-Wallis test comparing the different groups (Ctrl, Ko143, LPV and IVT). (B) Representative histograms of “5D3 shift” assay: Ko143 (1 µM), lopinavir (100 µM) and ivermectin (100 µM) conditions. (C) mRNA expression levels quantified by qPCR. (D) Protein expression levels quantified by western blot. (E) Representative image of western blot assay.

Although the efflux mediated by P-gp of hydrocortisone (Nakayama et al. 1999[40]), hydroxychloroquine (Weiss et al., 2020[68]), lopinavir (Agarwal et al., 2007[2]), dexamethasone (Ueda et al., 1992[61]), oseltamivir (Morimoto et al., 2008[37]), prednisolone (Karssen et al., 2002[29]) and ivermectin (Didier and Loor 1995[12]) has been described, we decided to evaluate the transport of all drugs by the MTT-based cell viability assay using parental and a cell line overexpressing P-gp. Ivermectin, dexamethasone and oseltamivir behaved as P-gp substrates, whereas hydrocortisone, prednisolone, lopinavir, hydroxychloroquine and chloroquine did not (see supplementary information Figure 3). Using the same approach for MRP1, we found that oseltamivir was the only drug not recognized as substrate. Lopinavir could be considered as strong substrate, whereas ivermectin could be considered as weak substrate (see supplementary information Figure 4). Ivermectin, chloroquine and dexamethasone have already been described as MRP1 substrates (Vezmar and Georges, 1998[66]; Ardelli, 2013[6]; Aberuyi et al., 2021[1]; Rendic, 2021[46]), while prednisolone and lopinavir have been described as non-substrates (Webster and Carlstedt-Duke, 2002[67]; Bierman et al., 2010[7]). In summary, our data confirmed that many drugs that were used for COVID-19 interact with the main ABC transporters involved in MDR, being inhibitors or substrates, and in some cases, the inhibitor is also transported, such as ivermectin on P-gp.

### Mechanism of ABCG2 inhibition exploited by in vitro approaches

Since lopinavir and ivermectin inhibited ABGC2, and the IC_50_ value of ABCG2 inhibition caused by lopinavir is in the same order of magnitude of the plasma concentration, the molecular mechanism of inhibition was studied. The interaction of drugs with ABC transporters can be studied by different *in vitro *approaches, including the use of conformational antibodies (Zattoni et al., 2022[71]). In the case of ABCG2 transporter, the antibody called clone 5D3 recognizes an extracellular epitope of the protein (Taylor et al., 2017[57]). In general, ABCG2 inhibitors induce a conformational change that increases the 5D3 binding, in contrast to ABCG2 substrates, which do not trigger this “5D3 shift” (Telbisz et al., 2012[59]; Zattoni et al., 2022[71]). Lopinavir and ivermectin induced an increase in 5D3 binding, such as the ABCG2 reference inhibitor Ko143 (Figure 4A(Fig. 4)). As shown by the histograms, the 5D3 shift is more pronounced for lopinavir and ivermectin than Ko143 (Figure 4B(Fig. 4)). This result agrees with our previous data that demonstrated that lopinavir and ivermectin are non-transported inhibitors of ABCG2 (Figures 2(Fig. 2) and 3(Fig. 3)).

Drugs that inhibit the transport activity of ABC transporters are called functional inhibitors. This inhibition effect is settled on the direct binding of ligands in a druggable binding pocket of these transporters, commonly located at transmembrane domains (Kowal et al., 2021[32]; Zattoni et al., 2022[71]). However, drugs targeting transcriptional or posttranslational protein levels have been described as promising to overcome the MDR phenotype. Drugs that trigger this effect are considered modulators, to differentiate functional inhibitors. In addition, a dual effect, direct transport inhibition and decreased protein levels are advantageous but poorly observed (Zattoni et al., 2022[71]). Here, the effect of lopinavir and ivermectin on ABCG2 was tested at transcriptional and translational levels, by qPCR and western blot, respectively. Considering a half-life of approximately 60 hours for ABCG2 in different cell lines (Imai et al., 2009[23]; Peng et al., 2010[42]), both assays were performed after 72 hours of drug exposure. In addition, to avoid a bias associated with the cytotoxicity effect, the highest drug concentration that does not decrease the cell viability was used to treat the cells. In this case, 6.25 and 1.56 µM for lopinavir and ivermectin, respectively (Figure 3D and E(Fig. 3)). As shown in Figure 4C(Fig. 4), lopinavir and ivermectin did not modulate the mRNA expression levels of ABCG2. The western blot results also revealed an absence of effect of both drugs on protein expression levels (Figure 4D(Fig. 4)). Together, these data confirm that lopinavir and ivermectin are not modulators of ABCG2 expression levels and should be classified as functional inhibitors of ABCG2.

**Figure 5:**
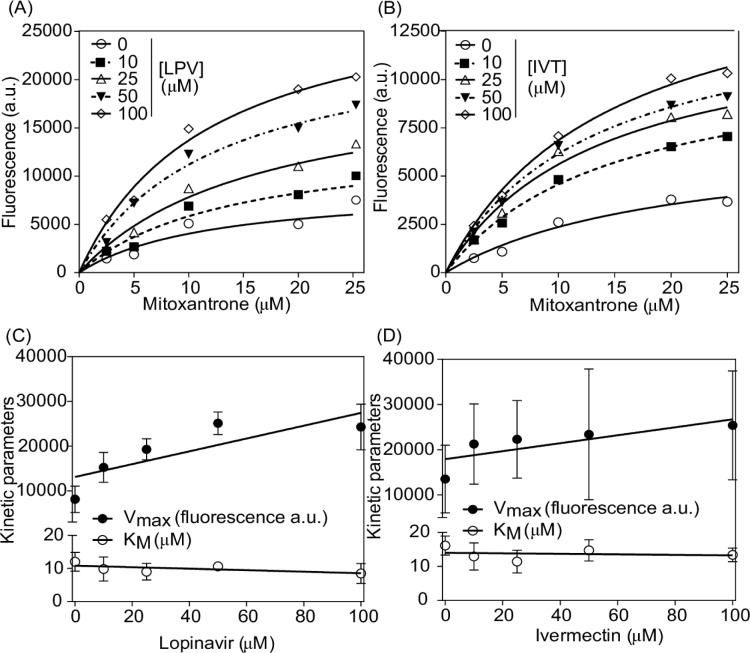
Kinetic behavior of mitoxantrone ABCG2-mediated efflux by flow cytometry. Intracellular fluorescence was determined using a range of mitoxantrone/inhibitors concentrations. (A) Lopinavir (LPV). (B) Ivermectin (IVT). Comparison of V_max_ and K_M_ of (C) lopinavir and (D) ivermectin. Data represent the mean ± SD of at least three independent experiments.

To get insights into the biochemical mechanism of ABCG2 inhibition, the type of inhibition caused by ivermectin and lopinavir was investigated by varying the concentrations of the inhibitor and the substrate mitoxantrone. This ABCG2 substrate was used instead of Hoechst 33342 because only with mitoxantrone a saturation curve was achieved (Figure 5A and C(Fig. 5)). Both drugs caused a noncompetitive inhibition since an increase of V_max_ with no effect on K_M_ value was observed (Figure 5B and D(Fig. 5)). The ABCG2 noncompetitive inhibition was already reported by some compounds, such as stilbene derivatives (Valdameri et al., 2012[64]). Recently, an uncompetitive and mixed-type of inhibition were also described for indenoindole (Guragossian et al., 2021[19]) and porphyrins, respectively (Zattoni et al., 2022[72]). 

### Molecular docking and dynamic simulations

**Figure 6:**
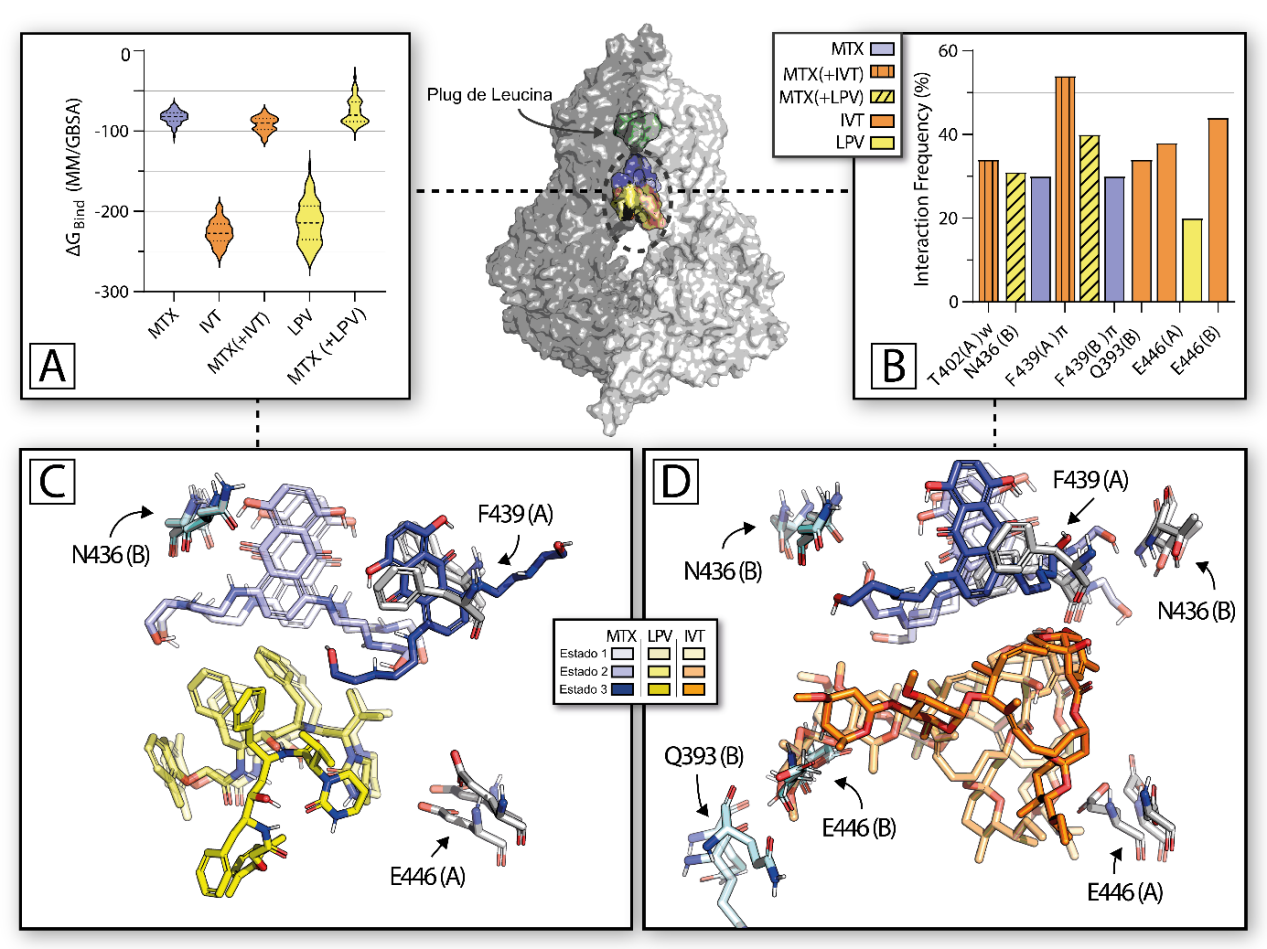
Molecular docking and molecular dynamics simulations. (A) The violin plot shows calculated binding affinity (kcal/mol) of MTX and MTX (+ inhibitor) complexes. (B) Bar chart, with interaction frequency (fraction of simulation time) between important residues and each molecule. (C and D) Sticks representation of three most prevalent populations of IVT (shades of orange), LPV (shades of yellow) and MTX (shades of blue). Residues with frequent interactions are indicated with arrows. Oxygen and nitrogen atoms are colored red and blue, respectively, and non-polar hydrogens are omitted. In the center: Surface representation of 6VXI and ligands in State 1, indicating the DBC, delimited by the leucine 554/555 plug (colored in pale green). MTX is mitoxantrone; LPV is lopinavir and IVT is ivermectin.

In order to gain further insights into the noncompetitive inhibition caused by lopinavir and ivermectin, *in silico* analyses were performed using molecular docking, molecular dynamics and MM/GBSA free binding energy calculations. Initially, molecular docking analysis showed a compatibility of the binding of inhibitors lopinavir and ivermectin in presence of the substrate mitoxantrone, revealing multiple conformations of each ligand co-occupying the drug binding cavity (DBC). The results suggested that the large DBC, located between both subunits of transmembrane helices, can accommodate one molecule of mitoxantrone and one molecule of each inhibitor simultaneously (Figure 6(Fig. 6)). Analysis of the docked complex showed that mitoxantrone occupied an “upper” position, closer to the L554/L555 plug, but ivermectin and lopinavir occupied a “below” position, closer of the cytoplasmic side (Figure 6(Fig. 6)). 

To further investigate the stability of the complex ligands-ABCG2 and specific types of interaction and other potential binding modes, molecular dynamics (MD) simulations were performed. The results were similar to those observed by docking (see supplementary information Figure 5). Mitoxantrone acted similarly in the presence or absence of an inhibitor, basically conserving the high frequency of F439/N436 interactions. The binding free energies (ΔG_bind_) estimated revealed that the binding of ivermectin or lopinavir does not significantly alter the mitoxantrone binding affinity (Figure 6A(Fig. 6)). Docking results initially showed that the most common interaction between ABCG2 transporter and mitoxantrone involves a π-stacking with F439 (Figure 6(Fig. 6)). MD results confirmed this observation and mitoxantrone binding was mainly favored by π-stacking interactions between the anthracenedione core of mitoxantrone and F439, as well by H-bonds with N436 from both subunits. The lack of interactions between mitoxantrone and ivermectin revealed by docking results was further elucidated by MD, which showed frequent water-mediated H-bonds present between the tetrahydropyran moiety of ivermectin and E446 (Figure 6B and C(Fig. 6)). This amino acid residue also interacted with lopinavir, indicating an important role in the stabilization of these inhibitors inside the drug pocket in presence of mitoxantrone. Together, these results suggest an absence of overlap between inhibitors and mitoxantrone, providing a plausible explanation for the noncompetitive inhibition. 

Furthermore, analysis of root-mean-square deviation (RMSD) values via a clustering algorithm showed three states in proximity for each inhibitor, indicating a low variation in terms of mobility inside the drug-binding pocket of ABCG2 (Figure 6C and D(Fig. 6)). These data suggest that binding of a second molecule, in this case, an inhibitor, cannot displace mitoxantrone from the DBC. In accordance, previous works of MD simulations using cryo-EM obtained structures strongly suggests that ligands can alter the protein conformation, allowing a simultaneous binding of substrates and inhibitors (Nagy et al., 2020[39]; Yu et al., 2021[70]).

Previous reports suggest that F439 is critical for the binding and stabilization of substrates by clamping the molecule inside the cavity (Gose et al., 2020[15]), and mutations at position 439 severely impair Hoechst 33342 and pheophorbide *a* (Gose et al., 2020[15]). Interestingly, the importance of N436 appears to be more specific and dependent on the molecule (Gose et al., 2020[15]; Kowal et al., 2021[32]), is indispensable for mitoxantrone transport (Guragossian et al., 2021[19]). The importance of E446 for ABCG2 function was also confirmed by mutagenesis studies (Khunweeraphong et al., 2017[30]; Szöllősi et al., 2019[55]). Our data suggest that mitoxantrone binds to the F436 region, while the inhibitors were trapped between E446 residues, blocking the proximity of transmembrane domains needed for the catalytic cycle of transport mediated by ABCG2. This spatial configuration allows these inhibitors to act as a wedge, preventing the approach of transmembrane helixes necessary to attain turnover conformations that potentially precede substrate efflux to the extracellular space (Yu et al., 2021[70]), but not avoiding substrate accommodation inside the DBC. However, a complete understanding of the catalytic cycle and drug dislocation from cavity 1 (drug binding pocket) to cavity 2 (above the L555), as well as the conformational changes induced by these inhibitors require additional studies. 

## Conclusion

Our results confirmed that lopinavir and ivermectin are functional inhibitors of ABCG2, P-gp and MRP1. Here, for the first time, we characterize the molecular mechanism of ABCG2 inhibition by lopinavir and ivermectin using cell-based and *in silico* approaches. Both drugs were not recognized as substrates and did not affect the mRNA and protein expression levels. In addition, lopinavir and ivermectin increased the binding of antibody 5D3, such as the reference inhibitor Ko143. Both compounds caused a noncompetitive inhibition, binding in a different site than mitoxantrone, as also confirmed by molecular docking, dynamic simulations followed by free energy calculations. Thus, the data shows that lopinavir is a potent ABCG2 inhibitor at maximum plasma concentrations and both lopinavir and ivermectin can be used as scaffolds for the design more powerful ABCG2 inhibitors.

## Declaration

### Acknowledgment

We thank Dr Attilio Di Pietro from IBCP (France), Dr Robert W. Robey and Dr Michael Gottesman from NCI/NIH (USA) for having provided the cell lines. This work was supported by PROIND 2020 (UFPR), Fundação Araucária/PPSUS (grant number 2020131000003) and Coordenação de Aperfeiçoamento de Pessoal de Nível Superior - Brasil (CAPES) (Finance code 001). The authors would like to thank the Finnish IT center for science (CSC), Ltd. for the generous computational resources. The study was financially supported by the TüCAD2, a program funded by the Federal Ministry of Education and Research (BMBF) and the Baden-Württemberg Ministry of Science as part of the Excellence Strategy of the German Federal and State Governments (TK). In addition, TK was funded by the Fortüne grant initiative under the Excellence Strategy.

### Declaration of competing interest

The authors declare that they have no conflict of interest.

## Supplementary Material

Supplementary information

## Figures and Tables

**Table 1:**
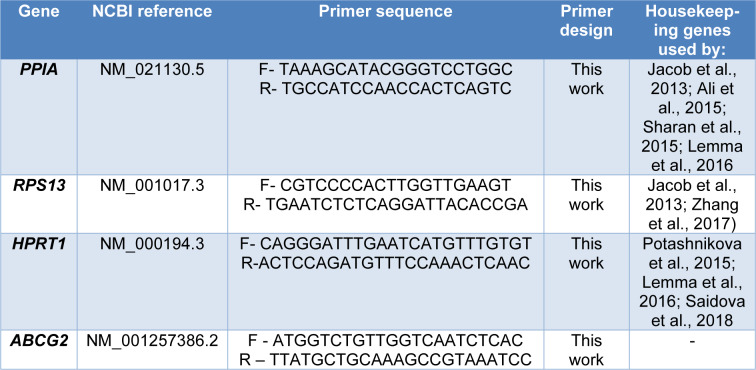
Nucleotide sequences of primers used for RT-qPCR
