# Supplementary material for: Structural and molecular characterization of lopinavir and ivermectin as breast cancer resistance protein (BCRP/ABCG2) inhibitors
Source: EXCLI J. 2023 Nov 14;22:1155–72. doi: 10.17179/excli2023-6427 (PMC10776880; doi:10.17179/excli2023-6427)
Supplement: Supplementary information [file EXCLI-22-1155-s-001.pdf]

EXCLI Journal 2023;22:1155-1172 – ISSN 1611-2156
Received: August 03, 2023, accepted: November 02, 2023, published: November 14, 2023

Supplementary information to:

Original article:

STRUCTURAL AND MOLECULAR CHARACTERIZATION OF LOPINAVIR AND IVERMECTIN AS BREAST CANCER RESISTANCE PROTEIN (BCRP/ABCG2) INHIBITORS

Julia de Paula Dutra[1], Gustavo Scheiffer[1], Thales Kronenberger[2,3], Lucas Julian Cruz Gomes[1], Isadora Zanzarini[1], Kelly Karoline dos Santos[1], Arun K. Tonduru[2], Antti Poso[2,3], Fabiane Gomes de Moraes Rego[4], Geraldo Picheth[4], Glaucio Valdameri[1], Vivian Rotuno Moure[1*]

1 Graduate Program in Pharmaceutical Sciences, Laboratory of Cancer Drug Resistance, Federal University of Parana, Curitiba, PR, Brazil
2 School of Pharmacy, Faculty of Health Sciences, University of Eastern Finland, P.O. Box 1627, FI-70211 Kuopio, Finland
3 (a) Department of Internal Medicine VIII, University Hospital Tuebingen, Otfried-Müller-Strasse 14, Tuebingen DE 72076, Germany
(b) Department of Pharmaceutical and Medicinal Chemistry, Institute of Pharmaceutical Sciences, Eberhard-Karls-Universität, Tuebingen, Auf der Morgenstelle 8, 72076 Tuebingen, Germany
(c) Cluster of Excellence iFIT (EXC 2180) "Image-Guided and Functionally Instructed Tumor Therapies", University of Tuebingen, 72076 Tuebingen, Germany
(d) Tuebingen Center for Academic Drug Discovery & Development (TüCAD2), 72076 Tuebingen, Germany
4 Graduate Program in Pharmaceutical Sciences, Federal University of Parana, Curitiba, PR, Brazil

* **Corresponding author:** Vivian Rotuno Moure, Graduate Program in Pharmaceutical Sciences, Laboratory of Cancer Drug Resistance, Federal University of Parana, Curitiba, PR, Brazil. E-mail: vivian.moure@ufpr.br

https://dx.doi.org/10.17179/excli2023-6427

This is an Open Access article distributed under the terms of the Creative Commons Attribution License (http://creativecommons.org/licenses/by/4.0/).

EXCLI Journal 2023;22:1155-1172 – ISSN 1611-2156
Received: August 03, 2023, accepted: November 02, 2023, published: November 14, 2023

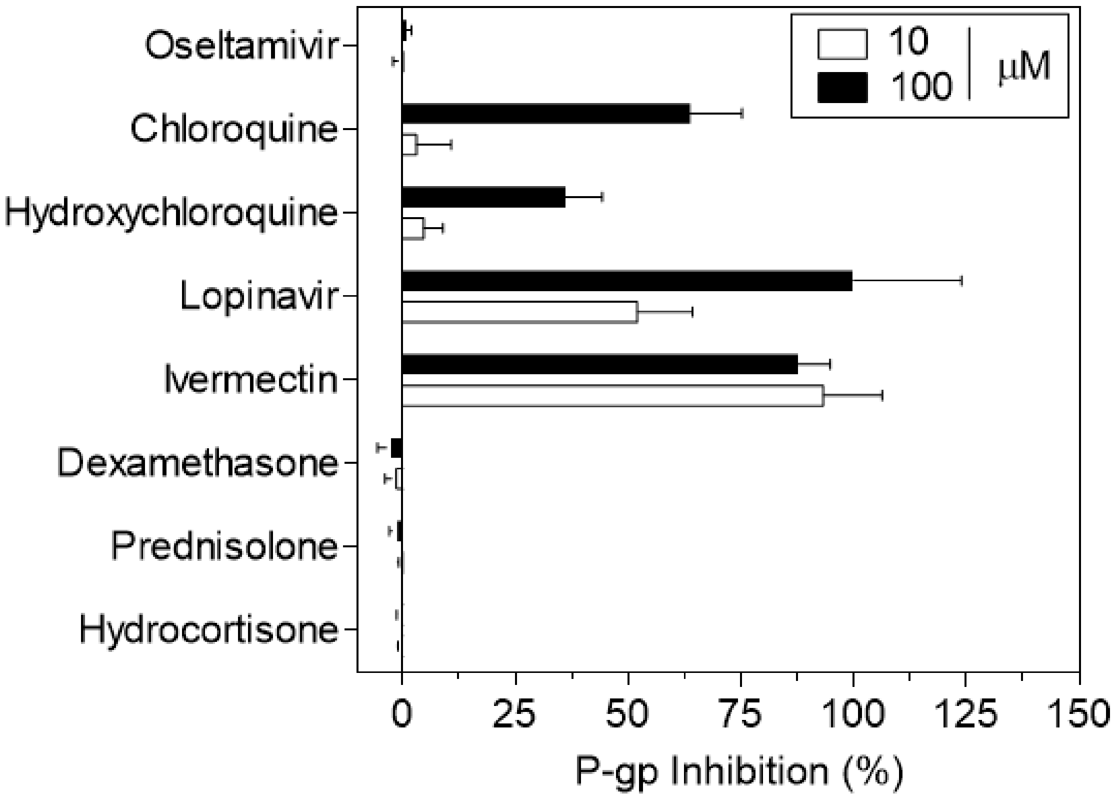

Supplementary Figure 1: Screening of drugs: hydrocortisone, prednisolone, dexamethasone, ivermectin, lopinavir, hydroxychloroquine, chloroquine and oseltamivir as P-gp inhibitors. Drugs were tested at 10 and 100 µM on NIH3T3-*ABCB1* cells by flow cytometry using rhodamine 123 at 10 µM as substrate. Elacridar (GF120918) at 1 µM was used as reference inhibitor (100% of inhibition). Data represents the mean ± SD of at least three independent experiments.

EXCLI Journal 2023;22:1155-1172 – ISSN 1611-2156
Received: August 03, 2023, accepted: November 02, 2023, published: November 14, 2023

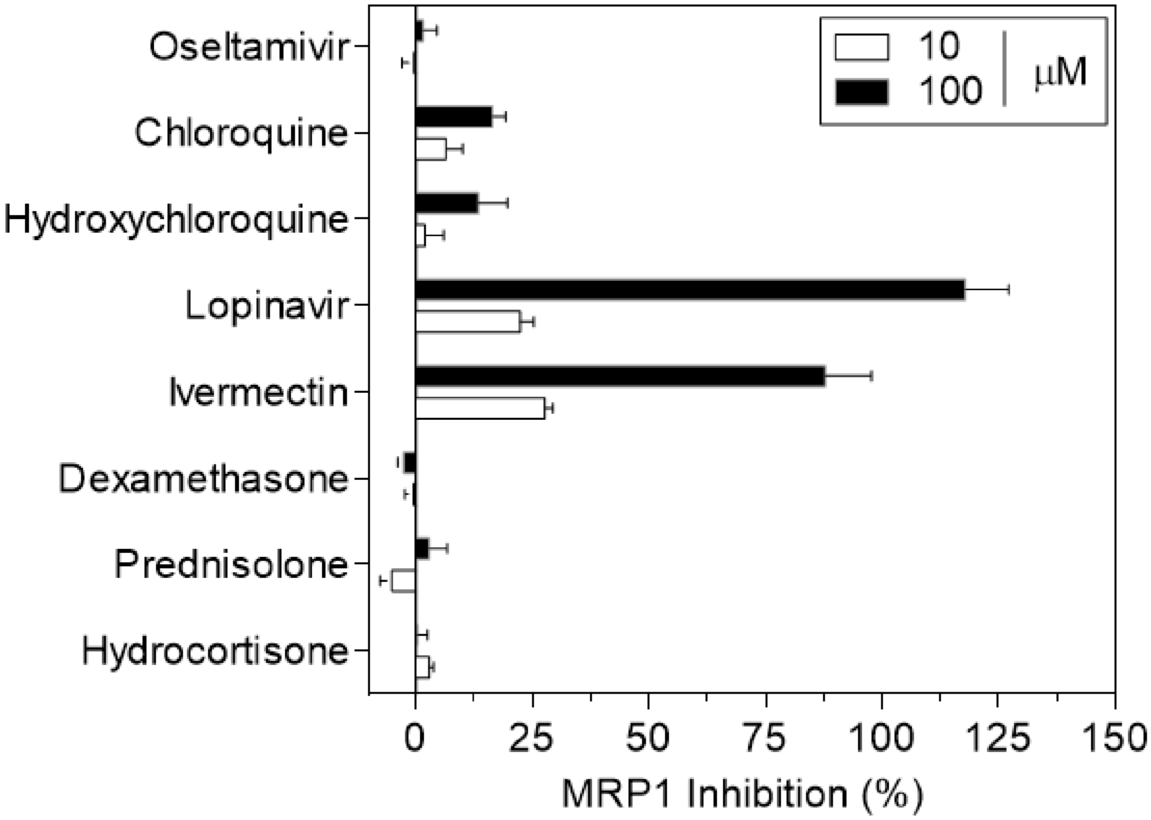

Supplementary Figure 2: Screening of drugs: hydrocortisone, prednisolone, dexamethasone, ivermectin, lopinavir, hydroxychloroquine, chloroquine and oseltamivir as MRP1 inhibitors. Drugs were tested at 10 and 100 µM on BHK21-*ABCC1* cells by flow cytometry using calcein-AM at 0.15 µM as substrate. BHK21 was used as control (100 % of inhibition). Data represents the mean ± SD of at least three independent experiments.

Supplementary Table 1: Therapeutic ratio (TR) values. TR corresponds to the ratio between IG_{50} and IC_{50} values. The IC_{50} values (concentration giving a half-maximal inhibition) of ABCG2 inhibition were obtained from Figures 1C and D. The IG_{50} values (concentration required to reduce cell viability by 50 %) of cytotoxicity were obtained from Figures 2D and E.

Compound	IC_{50} (µM) - inhibition	IG_{50} (µM) - cytotoxicity	TR (IG_{50}/IC_{50})
**Lopinavir**	23.4	14.98	0.64
**Ivermectin**	25.5	6.63	0.26

EXCLI Journal 2023;22:1155-1172 – ISSN 1611-2156
Received: August 03, 2023, accepted: November 02, 2023, published: November 14, 2023

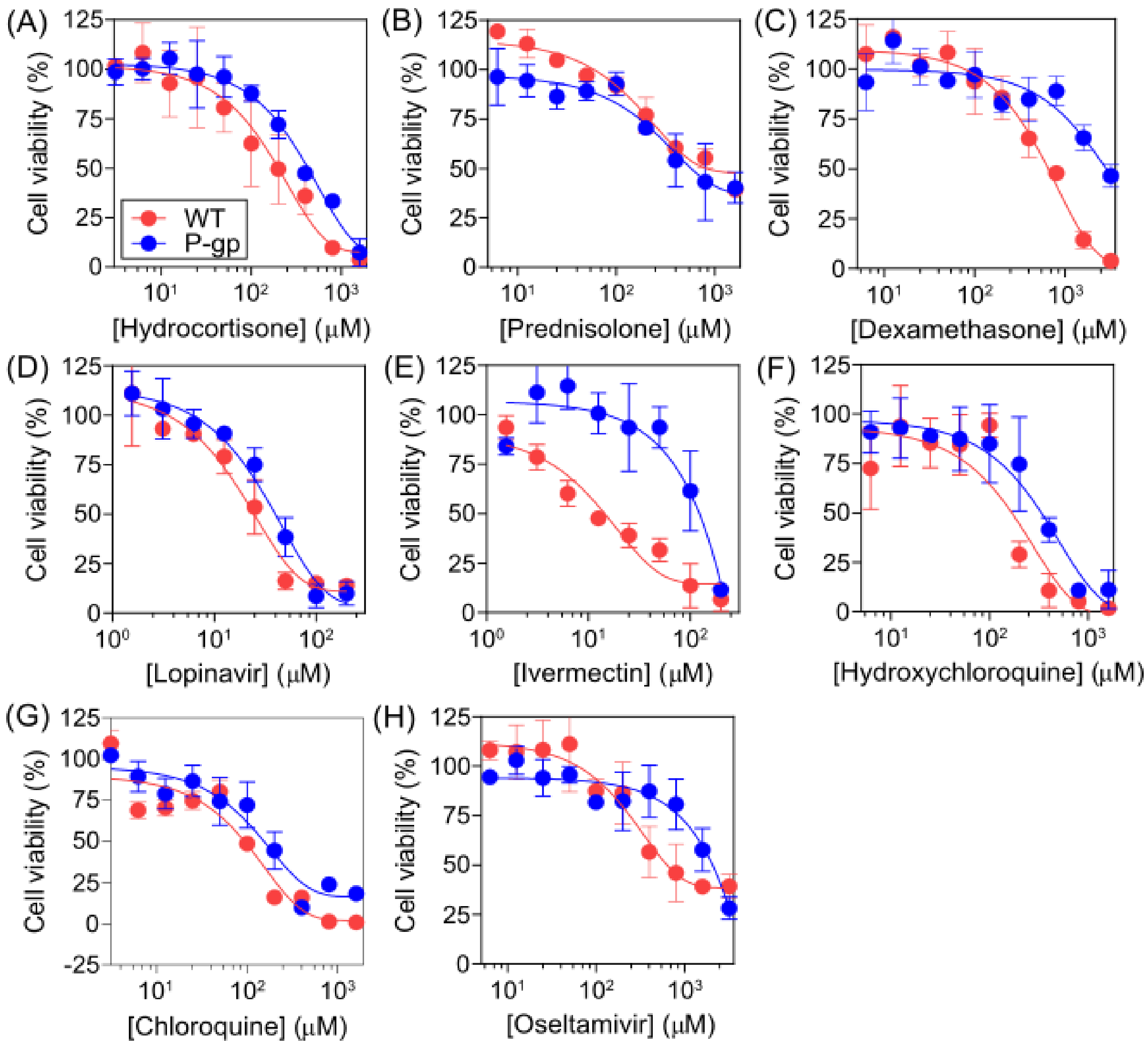

Supplementary Figure 3: Cytotoxicity and absence of transport mediated by P-gp transporter. MTT cell viability assay was performed on NIH3T3 and NIH3T3-*ABCB1* cells after 72 hours of treatment with (**A**) hydrocortisone, (**B**) prednisolone, (**C**) dexamethasone, (**D**) lopinavir, (**E**) ivermectin, (**F**) hydroxychloroquine, (**G**) chloroquine and (**H**) oseltamivir. Drugs were tested at different concentrations, as indicated in the graphs and the data represents the mean ± SD of at least three independent experiments. Cells treated with the vehicle (DMSO or H_2O) were considered as 100 % of viable cells.

EXCLI Journal 2023;22:1155-1172 – ISSN 1611-2156
Received: August 03, 2023, accepted: November 02, 2023, published: November 14, 2023

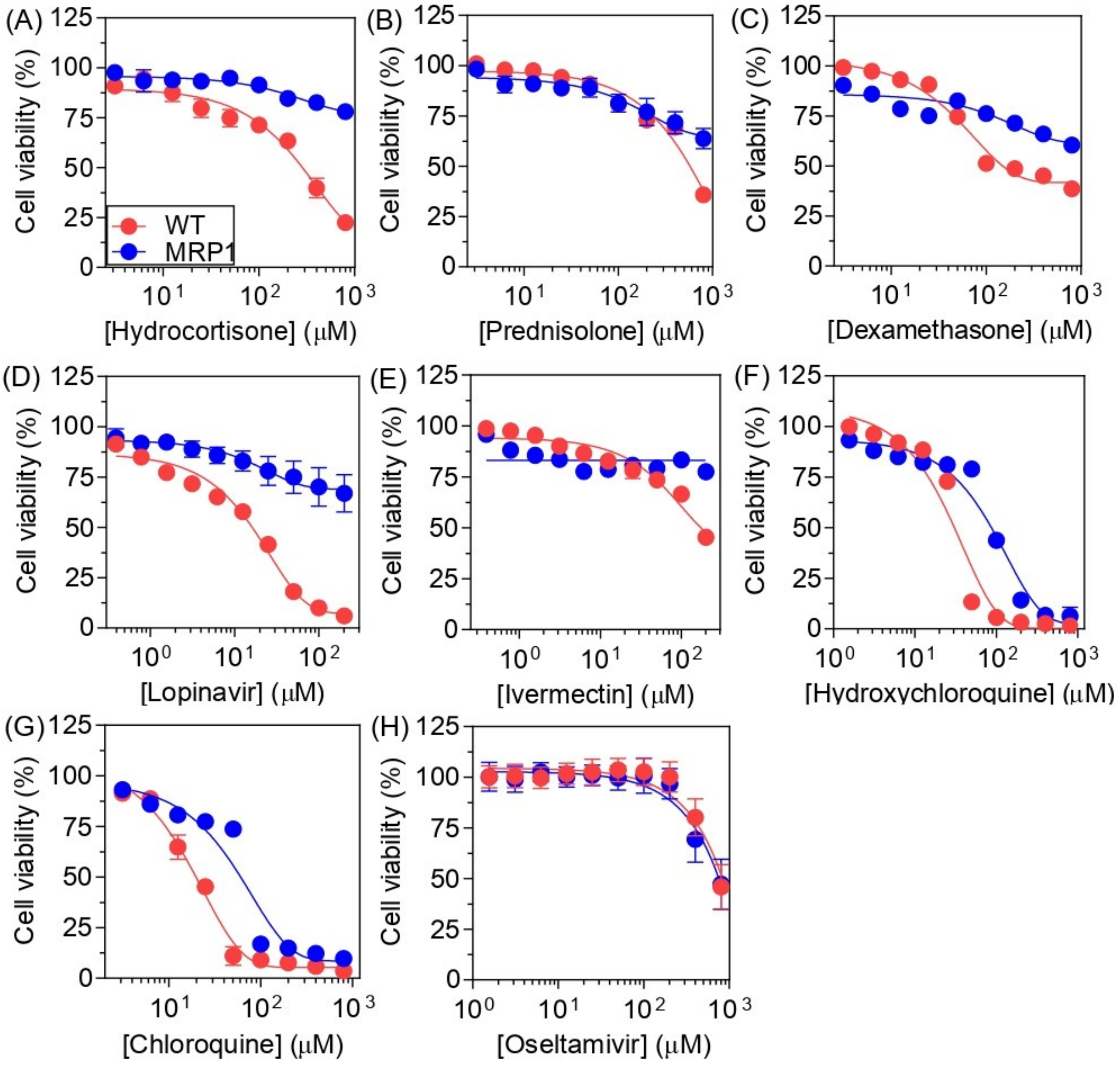

Supplementary Figure 4: Cytotoxicity and absence of transport mediated by MRP1 transporter. MTT cell viability assay was performed on BHK21 and BHK21-*ABCC1* cells after 72 hours of treatment with (**A**) hydrocortisone, (**B**) prednisolone, (**C**) dexamethasone, (**D**) lopinavir, (**E**) ivermectin, (**F**) hydroxychloroquine, (**G**) chloroquine and (**H**) oseltamivir. Drugs were tested at different concentrations, as indicated in the graphs and the data represents the mean ± SD of at least three independent experiments. Cells treated with the vehicle (DMSO or H_2O) were considered as 100 % of viable cells.

EXCLI Journal 2023;22:1155-1172 – ISSN 1611-2156
Received: August 03, 2023, accepted: November 02, 2023, published: November 14, 2023

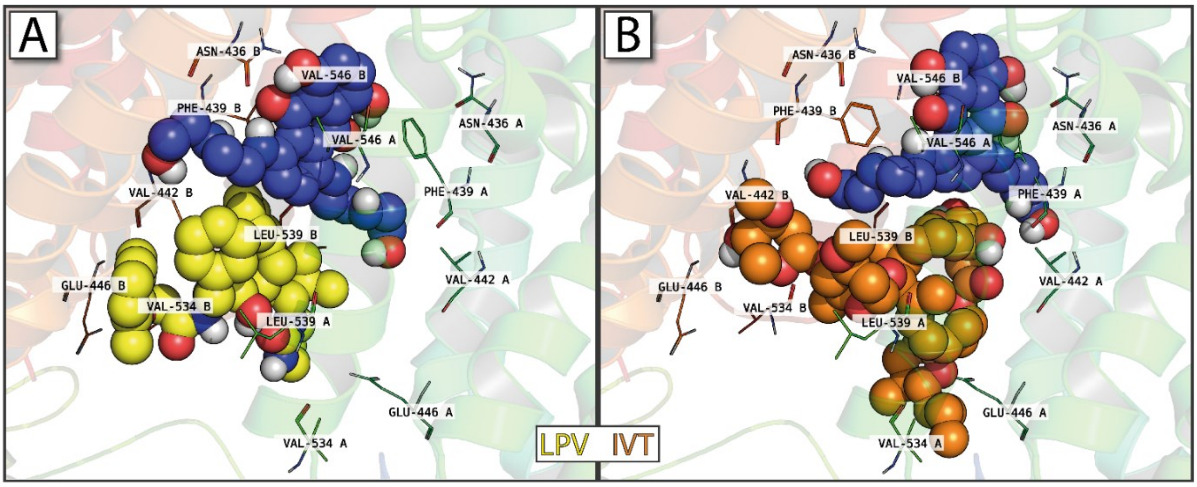

Supplementary Figure 5: Molecular Dynamics (MD) simulations: Mitoxantrone (MTX) colored in navy blue, lopinavir (LPV) in yellow and ivermectin (IVT) in orange are represented in spheres. The interaction of the amino acid side chains is represented in sticks and the protein main chains in cartoon. Oxygen atoms are in red, nitrogen in blue and hydrogen in gray. (**A**) Representative frame of MTX-LPV and (**B**) MTX – IVT. The molecules bind inside the central cavity of ABCG2 (PDB ID: 6VXI). MTX is mainly stabilized through hydrophobic interactions with PHE439 and H-bonds with ASN436. GLU446 plays a role on LPV and IVT bonding, both interfacing directly with MTX alkyl amino chains.
